# Anti-Proliferative Effect of Cytohesin Inhibition in Gefitinib-Resistant Lung Cancer Cells

**DOI:** 10.1371/journal.pone.0041179

**Published:** 2012-07-18

**Authors:** Anke Bill, Anton Schmitz, Katharina König, Lukas C. Heukamp, Jeffrey S. Hannam, Michael Famulok

**Affiliations:** 1 Chemical Biology and Medicinal Chemistry Unit, Life and Medical Sciences (LIMES) Institute, Rheinische Friedrich-Wilhelms-Universität Bonn, Bonn, Germany; 2 Institute of Pathology, University of Cologne, Köln, Germany; BioScience Project, United States of America

## Abstract

Epidermal growth factor receptor (EGFR) tyrosine kinase inhibitors (TKI), such as gefitinib, have been proven to efficiently inhibit the proliferation of a subset of non small-cell lung cancers (NSCLC). Unfortunately, the majority of NSCLC expressing wild type EGFR is primarily resistant to EGFR-TKI treatment. Here, we show that the proliferation of the gefitinib-resistant NSCLC cell lines H460 and A549 is reduced by the small molecule SecinH3 which indirectly attenuates EGFR activation by inhibition of cytohesins, a class of recently discovered cytoplasmic EGFR activators. SecinH3 and gefitinib showed a synergistic antiproliferative effect, which correlated with a profound inhibition of Akt activation and survivin expression. Treating mice bearing H460 xenografts with SecinH3 showed the antiproliferative and pro-apoptotic effect of SecinH3 in vivo. Our data suggest that targeting the EGFR indirectly by inhibiting its cytoplasmic activators, the cytohesins, has the potential to improve the treatment of primarily EGFR-TKI resistant lung cancers.

## Introduction

The introduction of epidermal growth factor receptor (EGFR) tyrosine kinase inhibitors (TKI) into the therapy of non small-cell lung cancer (NSCLC) bearing activating mutations of the EGFR has shifted the treatment paradigm from a chemotherapeutic to a targeted approach. Unfortunately, only 20 percent of adenocarcinomas of the lung bear activating mutations of EGFR and are responsive to EGFR-targeted therapy. Furthermore, patients under EGFR-targeted TKI therapy develop secondary resistance during therapy. Mutations in the EGFR play a decisive role in the response by the tumor to EGFR-targeted therapy. Activating mutations, in particular in exons 19 and 21, are predictive for a favorable initial response to EGFR-TKIs [1], [2], [3]. On the contrary, mutation of the so-called gatekeeper position in the ATP binding pocket of the EGFR kinase domain, i.e. substitution of threonine 790 by methionine, renders the cells resistant [4], [5], [6]. The gatekeeper mutation is the most common cause for the development of secondary resistance of responsive tumors. The majority of NSCLCs express wild-type EGFR and are, therefore, primarily resistant to EGFR-TKIs [7]. About 25% of these NSCLCs bear a mutated form of the Ras proto-oncogene, KRas G61H or G12V, and the presence of this mutation is an almost unmistakable indicator of resistance to EGFR-targeted therapy [8]. Nevertheless, in vitro studies using siRNA-mediated knock-down of the EGFR indicate that the proliferation of NSCLC cells expressing wild-type EGFR and bearing mutated KRas is still dependent to some extent on the EGFR [9], [10], [11], [12] suggesting that EGFR-TKI resistant cells are not totally independent of the EGFR and that, therefore, targeting the EGFR by means other than TKIs might lead to reduced proliferation even in EGFR-TKI resistant cells. Here, we show that treatment with SecinH3 of NSCLC cell lines expressing wild-type EGFR attenuates EGFR activation and signaling, reduces the proliferation of the cells in vitro and in vivo, and renders them responsive to the EGFR-TKI gefitinib. As SecinH3 inhibits cytoplasmic EGFR activators of the cytohesin family [13] our data suggest that targeting the EGFR indirectly by inhibition of its activators may represent a promising approach for developing EGFR-targeted therapies for the majority of NSCLCs which do not express mutant EGFR.

## Materials and Methods

### Materials

SecinH3, Secin16 and XH1009 were synthesized as described [14], [15], gefitinib was bought from Biaffin. H460 and A549 cells were from ATCC and cultivated in RPMI1640 (PAA) with 10% fetal calf serum (Lonza). The identity of the cell lines was verified at the end of the experimental period based on microsatellite genotyping by the ECACC Cell Line Identity Verification Service. The STR profiles matched the profiles of the cell lines as deposited in the ATCC and ECACC STR databases.

### Proliferation Assay

3×10^3^ cells per 96well were seeded into a clear, flat bottom 96well plate (TPP). After 24 h the cells were treated with the indicated concentrations of the inhibitors or solvent (final DMSO concentration 0.4%) in RPMI containing 50 ng/ml EGF or IGF-1 (Peprotech), respectively. Medium was changed daily for 3 days and cell proliferation was analyzed with a 3-(4,5-dimethylthiazol-2-yl)-2,5-diphenyltetrazolium bromide (MTT) assay (Promega) as described in the manufacturer’s protocol using a Varioscan microplate reader (Thermo Scientific). All assays were performed at least in triplicates. For calculation of the relative proliferation rate, the mean absorbance in the DMSO-treated cells was set as 1.

### Colony Formation Assay

Clonogenic growth assays were performed as described [16]. Briefly, 3000 cells/well were seeded into six-well plates, allowed to adhere over night and treated with the indicated concentrations of compound or DMSO for 72 h. Cells were dislodged, replated in six-well plates and cultured for 7 to 10 days in normal growth media. Colonies were stained with 0.1% Coomassie, 10% acetic acid, 30% methanol in PBS and analyzed using an Odyssey near-infrared scanner (LI-COR Biosciences).

### Tumor Xenograft

All animal procedures were in accordance with the German Laws for Animal Protection and were approved by the local animal protection committee and the local authorities (Bezirksregierung Köln, Germany). Tumors were generated by s. c. injections of 5×10^6^ H460 cells into nu/nu athymic male mice. After tumor establishment mice were randomized into two groups, control (vehicle) and SecinH3-treated mice. Mice were treated by daily i. p. injections (100 µl 2.5 mM SecinH3 in 50% DMSO/50% isotonic glucose solution or vehicle only). Tumor volume was measured daily and calculated by using the formula:
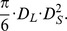



*D_L_*  =  larger diameter; *D_S_*  =  smaller diameter.

The TUNEL assay was performed according to the manufacturer’s manual (ApopTag Plus Peroxidase In Situ Apoptosis Kit, Merck Millipore).

Cleaved caspase 3 expression was determined by immunohistochemistry on formalin fixed and paraffin embedded tissue sections using an antibody specific for cleaved caspase 3 (1∶750, Cell Signaling Technology) after antigen retrieval at pH 6 as described [17].

**Figure 1:**
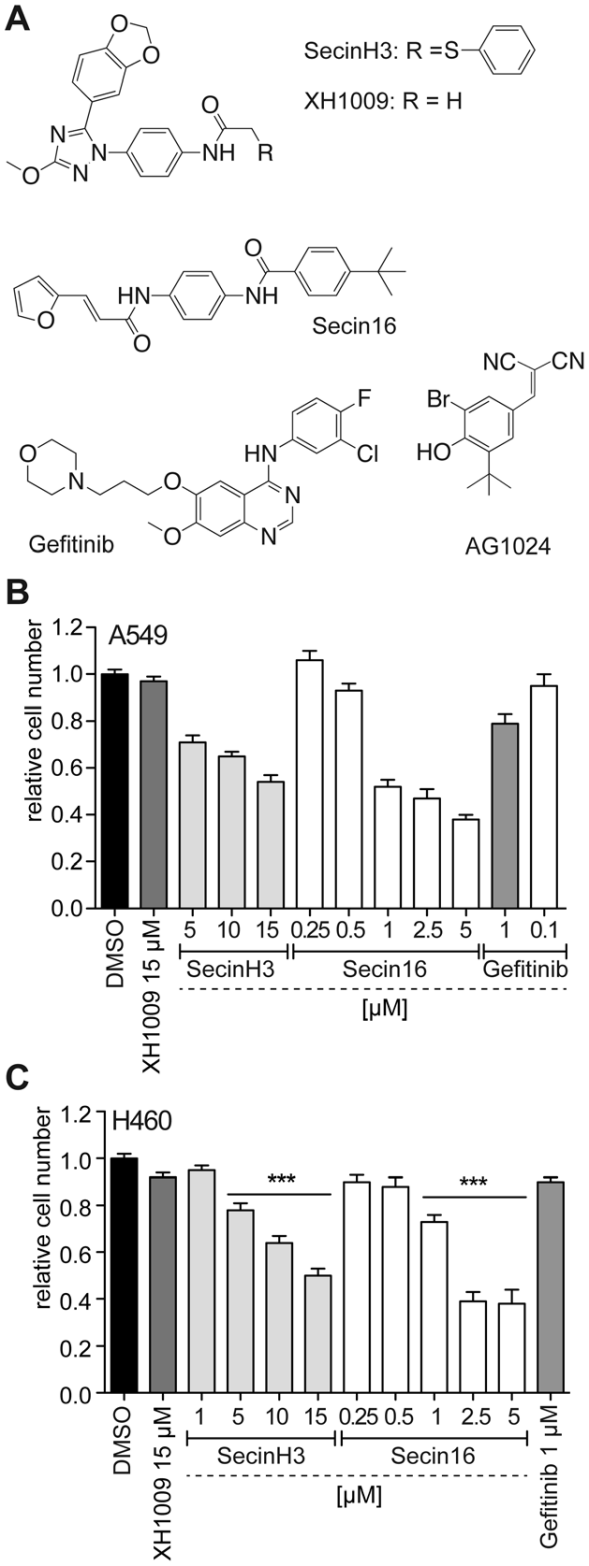
SecinH3 reduces proliferation of lung cancer cells expressing wild-type EGFR. A, structures of the inhibitors used in this study. B – C, proliferation of A549 (B) and H460 (C) cells was determined by MTT assay in the presence of the indicated inhibitors. ***, p<0.001 relative to DMSO-treated cells.

### Immunoblots

Cells were serum-starved overnight in the presence of the indicated inhibitor or DMSO (final DMSO concentration 0.4%). The medium and inhibitors were refreshed 1 h prior to stimulation. Stimulation was for 5 min with 50 ng/ml EGF or IGF-1, respectively. Cells were lysed in lysis buffer (20 mM Tris-Cl, pH 7.5/150 mM NaCl/1 mM EDTA/1 mM EGTA/2.5 mM sodium pyrophosphate/1 mM β-glycerophosphate/1 mM sodium vanadate/1% Triton X-100) supplemented with protease-inhibitor-mix HP (Serva). Normalized amounts of protein were separated by 6% SDS-PAGE and transferred onto nitrocellulose. The following antibodies were used: pAkt(Thr473) (Cell Signaling Technology), pEGFR(Tyr1086) (Epitomics), pIRS1(Tyr612) (Life Technologies), EGFR, survivin, p27 (SantaCruz Biotechnology), Hsc70 (Enzo Life Sciences). Detection was performed on an Odyssey scanner using DyLight800-labeled secondary antibodies (Thermo Scientific).

**Figure 2:**
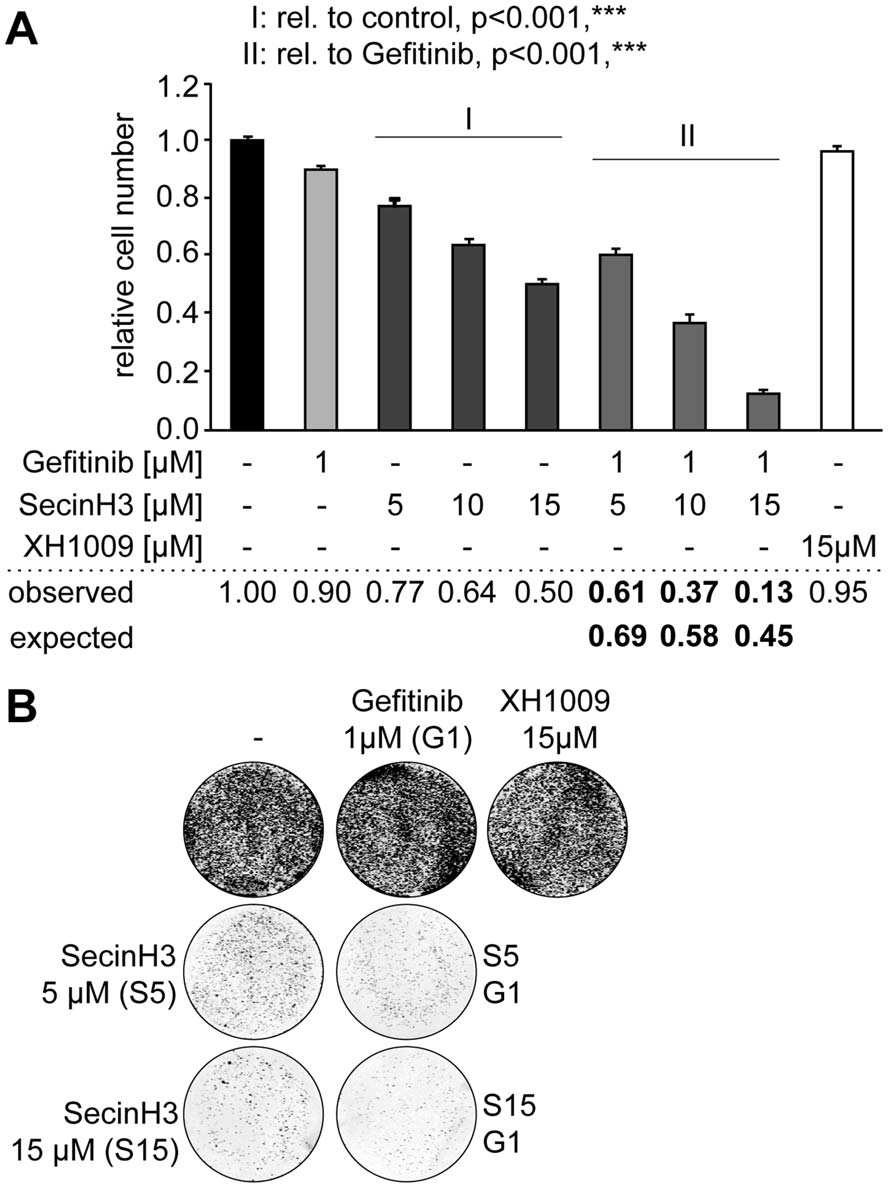
SecinH3 and gefitinib are synergistic. A – B, proliferation of H460 cells in the presence of gefitinib or SecinH3 alone or in their combination was determined by MTT assay (A) or by colony formation assay (B). In A the numbers give the relative value of proliferation with untreated cells set as 1. The proliferation assuming an additive effect of SecinH3 and gefitinib (expected) is compared to the value found in the experiments (observed). Observed values below the expected values indicate synergism.

**Figure 3:**
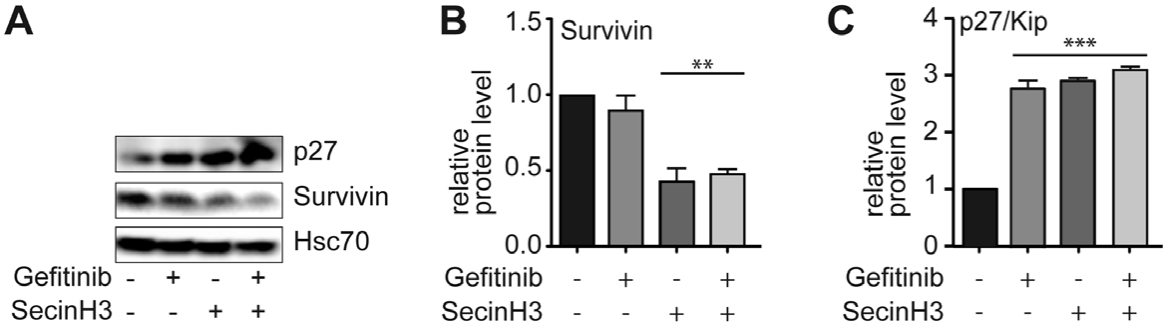
Survivin and p27/Kip expression. A – C, H460 cells were treated overnight with 1 µM gefitinib, 15 µM SecinH3 or their combination. A, the expression of p27Kip and survivin was analyzed by western blot. B, C, Statistical evaluation of the western blot data shown in A. ** in B indicates p<0.01 relative to DMSO-treated controls as well as to gefitinib-treated cells. *** in C indicates p<0.001 relative to DMSO-treated controls. n = 3.

**Figure 4:**
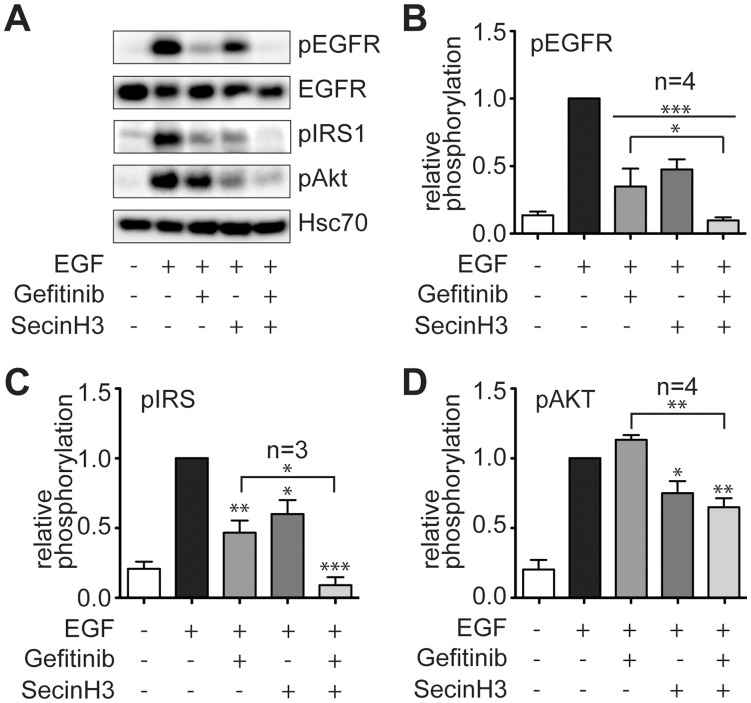
Effect of gefitinib and SecinH3 on EGFR signaling. A – D, serum-starved cells were stimulated with EGF for 5 min and probed by western blot for the activation of the indicated proteins. A, representative Western blot. B – D, Statistical evaluation of the phosphorylation of EGFR (B), IRS1 (C), and Akt (D) in EGF-stimulated H460 cells in presence of gefitinib, SecinH3, or a combination of both inhbitors. p indicates the phosphorylated, i.e. activated, form of the protein. The signals were normalized for the loading control Hsc70. The error bars give the standard error. *, p<0.05, **, p<0.01, ***, p<0.001 relative to EGF-treated cells except for those comparisons which are specified by brackets.

### Statistics

Results are given as the mean ± standard error of the mean (SEM). Statistical analyses were performed with Prism (GraphPad Software) applying one-way ANOVA with Tukeýs post-test for the in vitro experiments and the t-test for the xenografts. Differences of means were considered significant at a significance level of 0.05.

## Results

### NSCLC Cells Expressing Wild-type EGFR Respond to SecinH3

Although NSCLC cells expressing wild-type EGFR do not respond to clinically relevant concentrations of EGFR-TKIs their proliferation appears to depend to some degree on EGFR signaling as has been shown by EGFR knockdown experiments [9], [10], [11], [12]. Therefore, we asked whether the proliferation of the NSCLC cell line A549, which expresses wild-type EGFR and KRas with the activating mutation G12S was affected by treating the cells with SecinH3 (Fig. 1A). SecinH3 is a cytohesin inhibitor [14], [15] and does not inhibit the tyrosine kinase activity of the EGFR directly [13]. Cytohesins have long been known as guanine nucleotide exchange factors for small G proteins of the ARF family [18] but have recently been shown to contribute to EGFR activation [17]. The mechanism of cytohesin-mediated EGFR activation, which is as yet unknown in detail, involves direct interaction of cytohesins with the EGFR, is ARF-independent, and can be inhibited by SecinH3. When A549 cells were treated with SecinH3 or the related cytohesin inhibitor Secin16 [19] a concentration-dependent reduction in proliferation was observed (Fig. 1B). As compared to the highly EGFR-addicted PC9 cells which express an EGFR bearing a deletion in exon 19 [20], the proliferation of which was completely inhibited at 15 µM SecinH3 [17], the reduction in proliferation was less pronounced in A549 cells which is in accordance with the lesser dependence of the A549 cells on EGFR signaling. Inhibition was not seen in cells treated with the control compound XH1009 which is structurally related to SecinH3 but does not inhibit cytohesins [14]. Gefitinib used at the therapeutic concentration of 1 µM only marginally inhibited the proliferation of A549 cells. The activity of the used gefitinib was verified by treating the gefitinib-sensitive cell line PC9 [20] where 100 nM gefitinib inhibited cell proliferation almost completely (data not shown). To exclude that the effect of SecinH3 was restricted to A549 cells the experiments were repeated in H460 cells, a NSCLC cell line expressing also wild-type EGFR but a different KRas mutant, namely G61H (Fig. 1C). As in the A549 cells, 15 µM SecinH3 resulted in about 50% reduction of proliferation. This value fits reasonably well to the half-maximal inhibitory concentration of SecinH3 for the catalytic activity of purified cytohesins which is 10–12 µM [14].

### SecinH3 and Gefitinib are Synergistic

Having shown that SecinH3 attenuated the proliferation of gefitinib-resistant NSCLC cells we asked whether treatment with SecinH3 might render the cells responsive to gefitinib and, thus, act synergistically with this EGFR-TKI. Therefore, the proliferation of H460 cells was determined by a 3-(4,5-dimethylthiazol-2-yl)-2,5-diphenyl tetrazolium bromide (MTT) assay in the presence of 1 µM gefitinib and increasing concentrations of SecinH3 (Fig. 2A). For each combination the inhibition expected for Bliss independence of the two compounds was calculated by the fractional product method [21] and compared to the observed degree of inhibition [22]. For all three combinations tested, a synergistic effect of gefitinib and SecinH3 was found. The MTT assay determines proliferation indirectly by measuring the metabolic activity of the cell population. To gain additional and more direct proof for the inhibition of proliferation a colony formation assay was performed (Fig. 2B). Also in this assay, the combination of SecinH3 with gefitinib was more efficient than each inhibitor alone.

**Figure 5:**
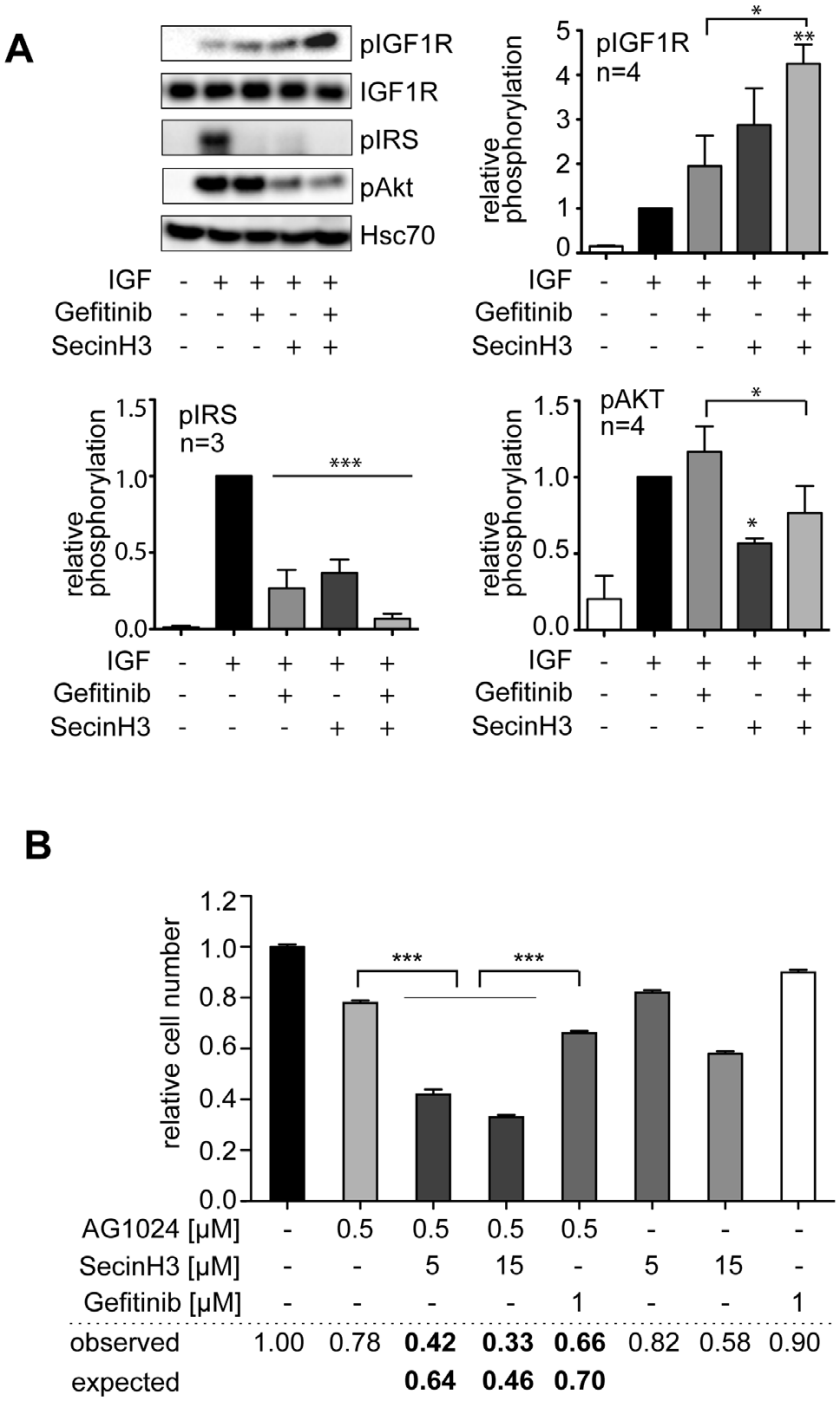
SecinH3 reduces IGF1R-dependent signaling and proliferation. A, H460 cells were serum-starved and treated overnight with 0.5 µM AG1024, 15 µM SecinH3 or their combination. After stimulation with IGF1 for 5 min the phosphorylation state of the indicated proteins was analyzed by western blot. The graphs summarize 3 experiments, the error bars give the standard error. *, p<0.05, **, p<0.01, ***, p<0.001 relative to IGF1-treated cells except for those comparisons which are specified by brackets. B, proliferation of H460 cells in the presence of the indicated inhibitors was determined by MTT assay. The numbers give the relative value of proliferation with untreated cells set as 1. The proliferation if an additive effect of SecinH3 and gefitinib is assumed (expected) is compared to the value found in the experiments (observed). Observed values below the expected values indicate synergism. ***, p<0.001.

Inhibition of EGFR signaling in EGFR-dependent cells is known to result in cell cycle arrest and to lead to the induction of apoptosis. As surrogate markers for cell cycle arrest and apoptosis, we determined the expression of the cell cycle progression inhibitor p27Kip1 and the apoptosis inhibitor survivin (Fig. 3A). Treatment of the cells with SecinH3 or gefitinib resulted in a decrease in survivin (Fig. 3B) and an increase in p27Kip1 (Fig. 3C), respectively. This effect was more pronounced in cells treated with both inhibitors.

As expression of both proteins, p27Kip1 and survivin, is regulated by Akt signaling the phosphorylation of Akt was analyzed in response to EGF stimulation of the cells (Fig. 4A). Whereas the EGF-induced phosphorylation of the EGFR and the adapter protein insulin-receptor substrate 1 (IRS1) was drastically reduced by gefitinib (Fig. 4B, C) the phosphorylation of Akt was not (Fig. 4D), indicating that Akt signaling was unchanged in gefitinib-treated H460 cells. In contrast, SecinH3 had a less inhibitory effect on EGFR and IRS1 phosphorylation but showed substantial inhibition on Akt activation. Consequently, the combination of SecinH3 and gefitinib resulted in almost complete inhibition of the phosphorylation of all three proteins. These data indicate that the antiproliferative effect of combined SecinH3/gefitinib treatment is due to efficient inhibition of the EGFR – Akt signaling axis.

**Figure 6:**
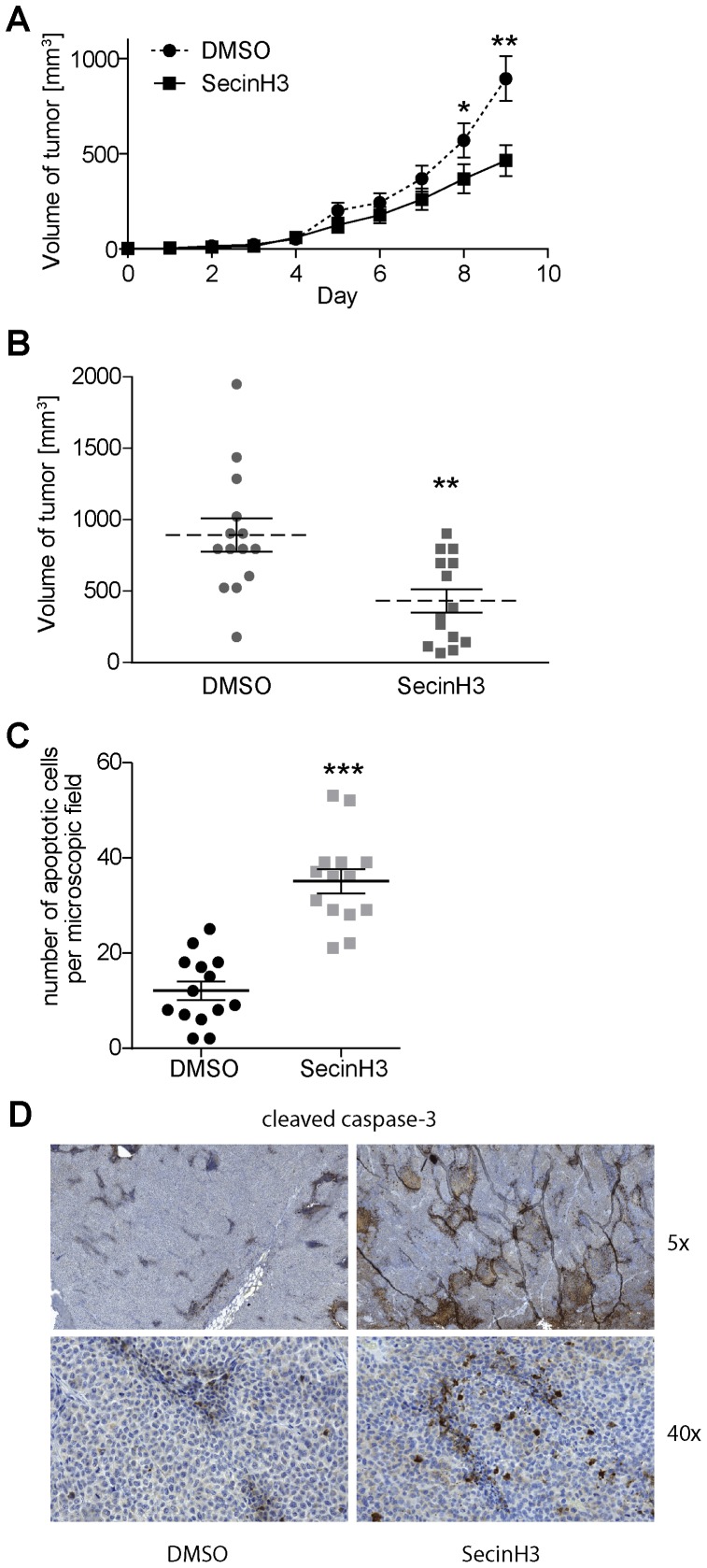
SecinH3 retards growth of H460 xenografts in vivo. Mice bearing subcutaneous H460 xenografts were treated with SecinH3 by daily intraperitoneal injections. A, tumor volume was determined each day. On day 9 the experiment had to be stopped because the tumor volume in the control animals exceeded 1 cm^3^. B, distribution of tumor volumes on day 9. C – D, the degree of apoptosis was analyzed in sections of tumors on day 9. C, the number of fragmented nuclei was determined by TUNEL staining. D, cleaved, i.e. activated, caspase-3 was detected by imunohistochemistry. Representative tumor sections are shown. 5× and 40× indicate the magnification of the objective. For all graphs, means and standard errors are shown (n = 14). *, p<0.05, **, p<0.01, ***, p<0.001.

### SecinH3 Reduces IGF1R-dependent Signaling and Proliferation

In addition to the EGFR, the insulin-like growth factor-1 receptor (IGF1R) is a major regulator of Akt activation and cross-talk between IGF1R and EGFR or EGFR family members such as Her2 has been reported as a mechanism of resistance against drugs targeting EGFR or Her2 in several types of cancers [16], [23], [24], [25], [26]. As SecinH3 inhibits signaling downstream of the insulin receptor [15], [27] which shares key features with IGF1R signaling we tested whether treatment of H460 cells with SecinH3 resulted in reduced activation of the IGF1R – Akt signaling pathway (Fig. 5A). In agreement with results obtained for the insulin receptor, the autophosphorylation of the IGF1R was not reduced upon SecinH3 treatment, but increased. Nevertheless, the activation of IRS1 and Akt was substantially decreased. Inhibition of Akt activation was not seen upon gefitinib treatment. The increase in IGF1R phosphorylation upon treatment with gefitinib and SecinH3 is in agreement with previous observations that inhibition of the EGFR results in increased activation of the IGF1R [16], [22], [25], [26]. We then asked whether SecinH3 reduced proliferation in the presence of IGF1 (Fig. 5B). Indeed, the proliferation was reduced upon SecinH3 treatment and the reduction was synergistic with the IGF1R inhibitor AG1024. The combination of gefitinib and AG1024 also showed a synergistic antiproliferative effect albeit less pronounced than the combination of SecinH3 and gefitinib.

### SecinH3 Retards Growth of H460 Xenografts in vivo

Having shown that SecinH3 reduces the proliferation of EGFR-wild type NSCLC cells in vitro we asked whether tumor growth would be inhibited also in vivo. Therefore, nude mice bearing subcutaneous H460 xenografts were treated by daily intraperitoneal injections of SecinH3. Treatment with SecinH3 significantly retarded tumor growth (Fig. 6A). When the mice were sacrificed after 9 days of treatment significantly smaller tumors were observed in the SecinH3-treated group as compared to the solvent-treated group (Fig. 6B). In histological sections of the tumors TUNEL staining revealed an increased rate of apoptosis in the treated animals (Fig. 6C), which was also evident by caspase-3 staining (Fig. 6D). In summary, our data show that SecinH3 in vitro and in vivo reduces the proliferation of NSCLC cells expressing wild-type EGFR.

## Discussion

In this report, we show that NSCLC cells displaying primary resistance against the EGFR-TKI gefitinib respond to treatment with SecinH3, which inhibits the EGFR indirectly by targeting its cytoplasmic activators of the cytohesin family. This result may appear unexpected because resistance against gefitinib may be interpreted as independence of EGFR signaling. This is, however, not the case. Treatment with EGFR-specific siRNAs of different EGFR-TKI resistant NSCLC cell lines expressing wild-type EGFR resulted in reduced proliferation of the cells [9], [10], [11], [12] indicating that EGFR-TKI resistance and EGFR independence are at least not always equivalent. Accordingly, it has been shown that the EGFR contributes to the survival of cancer cells independent from its kinase activity [28]. These data indicate that the kinase-inhibited EGFR is still able to activate signaling pathways which stimulate proliferation or inhibit cell death. In support of this assumption is the observation that inhibition of the EGFR by erlotinib induces heterodimerization of the EGFR with the IGF1R which activates the IGF1R and its signaling to downstream kinases including Akt [16]. Cytohesins have been shown to directly interact with the EGFR and thereby to influence its conformation [17]. As a particular conformation of the EGFR, the asymmetric dimer, is a prerequisite for kinase activation [29], the modulation of EGFR conformation has been implicated in the activation of the EGFR by cytohesins, a process which is inhibited by SecinH3. As the treatment of H460 cells with SecinH3 resulted in an increased phosphorylation of the IGF1R a similar function of cytohesins during the activation of the IGF1R by the EGFR can be excluded. Apparently, SecinH3 inhibits signaling by the EGFR-activated IGF1R downstream of the receptor. This finding is in full agreement with earlier observations that SecinH3 did not affect insulin receptor activation but inhibited signaling downstream of the receptor at the level of the adapter protein insulin receptor substrate-1 resulting in reduced Akt activation [15], [27], [30]. Thus, the anti-proliferative activity of SecinH3 in EGFR-TKI resistant NSCLC cells may be explained by its dual inhibitory effect on both, EGFR and IGF1R signaling, which may prevent the cells from circumventing EGFR inhibition by increased IGF1R signaling. We would like to point out that our data do not exclude the possibility that the synergistic anti-proliferative effect of SecinH3 with gefitinib may result from SecinH3 affecting signaling by RTKs in addition to the IGF1R. In particular, amplification of the hepatocyte growth-factor receptor c-MET [31] and the presence of the EML4-Alk fusion protein [32] have been related to EGFR-TKI resistance. The activation of these RTKs by cytohesins has, however, not yet been investigated and thus we do currently not know whether signaling by these RTKs might be affected by SecinH3.

Enhanced activation of the IGF1R has been recognized as a major cause of resistance against EGFR-targeted therapies in cancer cells of different origin. Accordingly, in vitro experiments showed combined inhibition of IGF1R and EGFR activity to reduce tumor cell proliferation synergistically [16], [23], [24], [25], [26]. Unfortunately, these results were not translated into clinical trials where, up to now, combination therapies applying EGFR and IGF1R targeting therapies failed to show significant improvements over single therapy [33], [34]. The reasons for these disappointing results are currently unknown but may lie in the incomplete understanding of the molecular details of the crosstalk between EGFR and IGF1R signaling which precludes rational selection of patients who may benefit from combination therapy. Mutations which predict response to targeted therapy, as for the EGFR, have not been reported for the IGF1R [35] and can, thus, not be used to select patients. In a clinical study searching for biomarkers predicting benefit from combined EGFR/IGF1R inhibition mutant KRas was identified [36]. In vitro studies showed, however, conflicting data on the antiproliferative effect of combined inhibition of the EGFR and the IGF1R in KRas-mutated H460 cells. Whereas one study did not find synergism [25] another study did [16]. In our study a weak synergism of gefitinib and AG1024 was found whereas stronger synergism was found for combinations of either of these compounds with SecinH3. These discrepancies highlight the notion that inhibiting identical targets by different inhibitors or inhibition strategies may lead to different biological outcomes. Hence, approaches which do not rely on direct inhibition of EGFR and IGF1R kinase activities but target these receptors indirectly via their activator or adaptor molecules may provide more complete understanding of these signaling pathways and thereby provide as yet unrecognized targets for novel therapeutic approaches.
